# Correction to “A MXene‐Based Nanothermal Knife Inhibits Aggresome‐Mediated Persister Formation for Preventing Dental Caries”

**DOI:** 10.1002/advs.77943

**Published:** 2026-09-28

Zhang, L. Yang, J. jiao, et al. “A MXene‐Based Nanothermal Knife Inhibits Aggresome‐Mediated Persister Formation for Preventing Dental Caries,” Adv. Sci. 12, no. 33 (2025): 12, e01501. https://doi.org/10.1002/advs.202501501

In Figure 9A, the pathological images of rat tongue mucosa for the **Control and Ti_3_C_2_+L+CHX** groups were inadvertently found to be duplicated. The image should be replaced with the correct version.


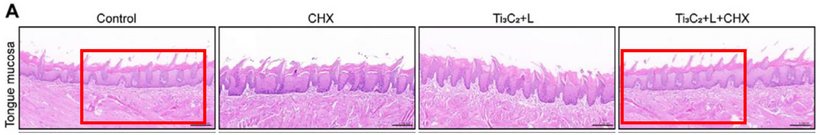


Original Figure 9A. The biocompatibility of Ti3C2‐mediated PTT in vivo. (A) H&E staining images for tongue mucosa and palate mucosa (scale bar, 100 µm).


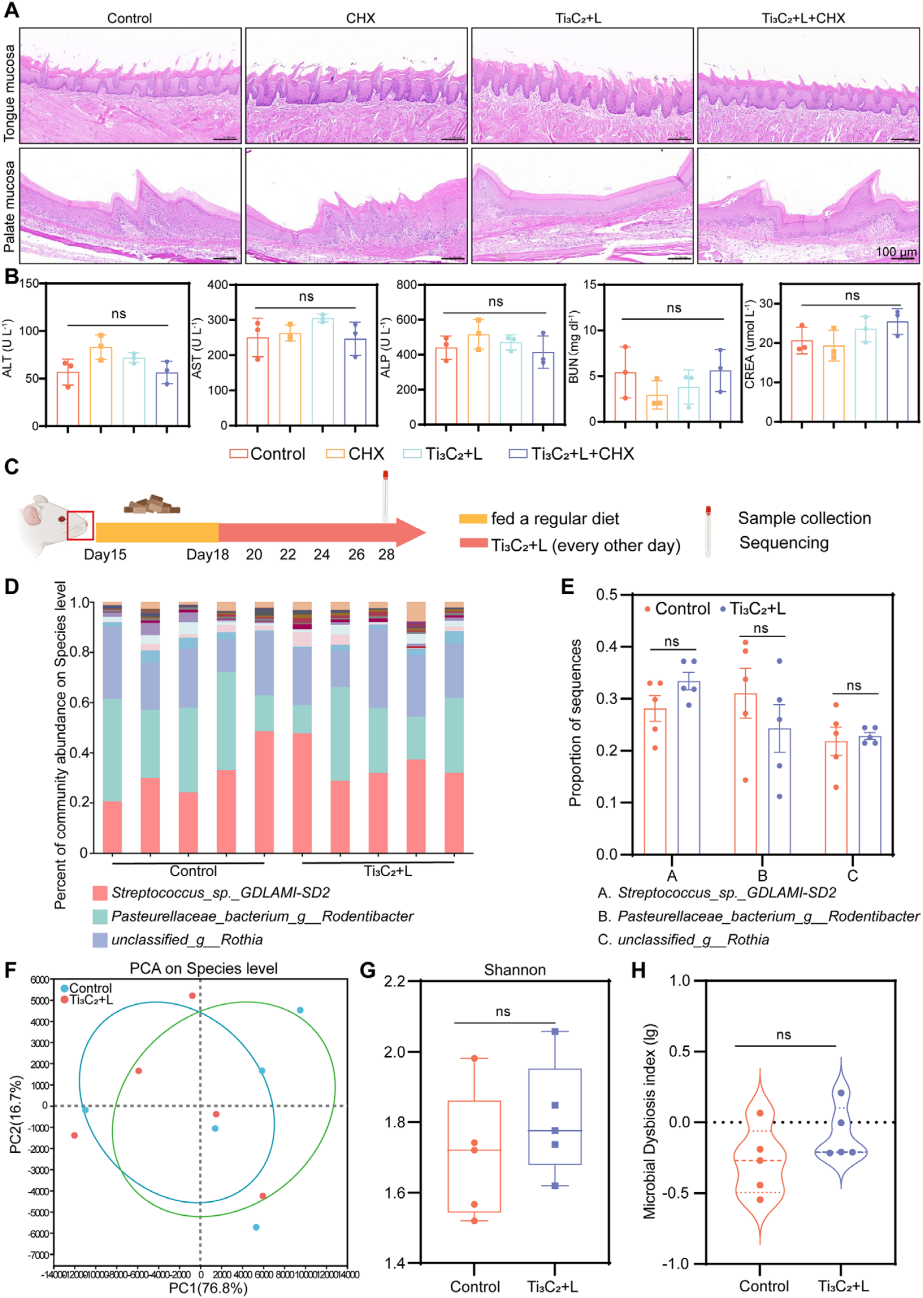


Corrected Figure 9A. The biocompatibility of Ti_3_C_2_‐mediated PTT in vivo. (A) H&E staining images of tongue mucosa and palate mucosa (scale bar, 100 µm). (B) Blood biochemistry analysis, testing alanine aminotransferase (ALT), aspartate aminotransferase (AST), alkaline phosphatase (ALP), blood urea nitrogen (BUN), and creatinine (CREA). (C) Schedule of rat model construction for testing the oral microbiota homeostasis after Ti_3_C_2_‐mediated PTT. (D) Bar plot of microbial community composition in the Control and Ti_3_C_2_+L groups. (E) Statistical testing for differences in dominant microbial populations between the Control and Ti_3_C_2_+L groups. (F) PCA shows the contrast between the Control and the Ti_3_C_2_+L groups based on the features of microbial communities. (G) Shannon index shows no noticeable differences between the Control and Ti_3_C_2_+L groups. (H) MDI plot showing the inter‐group differences in microbial communities at the genus level between the Control and Ti_3_C_2_+L groups (ns, *p* > 0.05).

We would like to emphasize that this correction does not affect the results, interpretation, or conclusions of the study. We apologize for this error.

